# Live viral measles-mumps-rubella revaccination and varicella vaccination in children and adolescents with juvenile idiopathic arthritis: a systematic review

**DOI:** 10.1007/s00431-026-07160-6

**Published:** 2026-06-16

**Authors:** Rosario Francesco Dipasquale, Paola Sinopoli, Alessia Mendicino, Romina Gallizzi

**Affiliations:** Pediatrics, Department of Health Sciences, University of Catanzaro “Magna Graecia”, 88100 Catanzaro, Italy

**Keywords:** Juvenile idiopathic arthritis, Live viral vaccines, Measles-mumps-rubella booster, Varicella vaccination, Immunogenicity, Vaccine safety

## Abstract

The purpose of this systematic review is to synthesize the available evidence on the immunogenicity and safety of live viral measles-mumps-rubella (MMR) booster/revaccination and varicella vaccination in children and adolescents with juvenile idiopathic arthritis. PubMed, Scopus, and Web of Science were systematically searched from inception to October 1, 2025, for studies including patients with juvenile idiopathic arthritis who had received live viral MMR or varicella vaccination and reporting immunogenicity, adverse events, vaccine-strain infection, breakthrough infection, or post-vaccination disease activity. Nine studies comprising 743 patients with juvenile idiopathic arthritis were included: five retrospective studies, three prospective cohort studies, and one open-label randomized controlled trial. Seven studies assessed MMR booster or revaccination, whereas two small prospective cohorts assessed varicella vaccination. MMR booster/revaccination was generally immunogenic and clinically safe in the short term, with no consistent increase in disease activity and no serious vaccine-related complications reported. However, 5-year seroprotection after MMR booster was lower among children receiving biologic disease-modifying antirheumatic drugs (DMARDs) at vaccination than among non-bDMARD users for measles (60% vs 86%), mumps (80% vs 94%), and rubella (60% vs 83%). Varicella vaccination induced protective antibodies in 82–83% of patients after two doses, but antibody levels were lower than those in healthy controls; VZV-specific cellular immunity was detectable in 72% in the larger cohort and appeared to persist longer than humoral immunity. No vaccine-strain varicella infection was reported, although mild breakthrough varicella occurred in some vaccinated patients.*Conclusion*: In clinically stable children with juvenile idiopathic arthritis, the available evidence supports the cautious use of MMR booster/revaccination and varicella vaccination under specialist supervision. The main residual concern is not a consistent safety signal but incomplete or less durable protection in selected biologic-treated patients, particularly for measles and rubella 5 years after MMR booster and for varicella in low responders. These findings support individualized vaccine timing and consideration of post-vaccination serology in selected higher-risk children.

**Table:** 

**What is Known:** • *Live viral vaccines in children with juvenile idiopathic arthritis are used cautiously because of concerns about attenuated-virus-related adverse events, disease flare, and reduced immunogenicity during immunosuppressive therapy.* • *The evidence base is stronger for MMR booster/revaccination than for varicella vaccination, because MMR data include one randomized trial and several larger cohorts, whereas varicella data come from two small prospective cohorts.*	
**What is New:** • *MMR booster/revaccination and varicella vaccination appeared generally safe in clinically stable children with juvenile idiopathic arthritis, with no serious vaccine-related complications or vaccine-strain infections reported in the included cohorts.* • *Five-year seroprotection after MMR booster was lower in biologic-treated children for measles, mumps, and rubella, supporting individualized vaccine timing and selected post-vaccination serologic monitoring.*

**Supplementary Information:**

The online version contains supplementary material available at 10.1007/s00431-026-07160-6.

## Introduction

Juvenile idiopathic arthritis (JIA) is the most common chronic rheumatic disease of childhood and a major cause of pain, functional impairment, and long-term morbidity [1]. Current management relies on non-steroidal anti-inflammatory drugs, intra-articular corticosteroids, methotrexate, and a growing range of biologic agents, which have substantially improved outcomes but may also modify host immunity status and vaccine responses [1, 2, 13].

Vaccination is a central component of preventive care in pediatric rheumatology, but live attenuated vaccines deserve separate consideration. Unlike inactivated vaccines, they may add concerns in immunosuppressed hosts, including reduced immunogenicity, reactivation of underlying disease, or attenuated-virus-related complications. For this reason, current recommendations support an individualized approach, with live attenuated vaccines considered mainly in clinically stable children assessed by pediatric rheumatology specialists [2, 3].

These concerns extend beyond disease flare alone. In daily pediatric rheumatology practice, clinicians must balance the theoretical risk of attenuated-virus-related adverse events against the risk of leaving immunosuppressed children susceptible to vaccine-preventable infections. This balance is increasingly relevant when community vaccination coverage is suboptimal, and herd immunity cannot be assumed to provide reliable indirect protection.

Although data on inactivated vaccines in JIA are generally reassuring, evidence specific to live attenuated vaccines remains more limited and methodologically heterogeneous [3].

The aim of this systematic review was therefore to summarize the evidence on the immunogenicity and safety of live viral MMR booster/revaccination and varicella vaccination in children and adolescents with juvenile idiopathic arthritis, with particular attention to vaccination context, seroprotection, durability of response, adverse events, vaccine-strain or breakthrough infections, and post-vaccination disease activity under immunomodulatory therapy.

## Materials and methods

### Eligibility criteria

Studies were eligible if they enrolled children or adolescents (≤ 19 years) with juvenile idiopathic arthritis, any subtype, and reported extractable data on live viral MMR or varicella vaccination administered as part of routine care or within the source study. Studies of broader pediatric autoimmune or inflammatory rheumatic disease populations were included only when JIA-specific data were extractable. Eligible vaccine contexts included MMR booster vaccination, MMR revaccination/catch-up vaccination, and varicella vaccination. When available, we extracted whether vaccination represented a primary series, booster, revaccination, or an unclear context based on the terminology and baseline vaccination history reported in the source article.

Eligible outcomes were immunogenicity outcomes, including seroprotection, seroconversion, antibody concentrations, durability of humoral response, and cellular immunity, and/or safety outcomes, including local or systemic adverse events, serious adverse events, vaccine-strain infection, breakthrough infection, disease flares, and changes in validated disease activity measures. Eligible comparators included healthy controls, JIA controls, treatment subgroups, pre/post comparisons, or no comparator.

Exclusion criteria were (1) absence of measurable or objective outcome variables; (2) cross-sectional design without post-vaccination outcome assessment; (3) animal studies; (4) non-English language; (5) non-peer-reviewed studies, including posters and conference abstracts; (6) studies with fewer than five patients and no immunologic outcomes; and (7) communications, correspondence, and editorials.

### Search strategy

PubMed, Scopus, and Web of Science were systematically searched from inception to October 1, 2025. The complete database-specific search strategies are reported in Supplementary Table S1. In addition, the reference lists of previous systematic reviews on related topics were manually screened. This systematic review was conducted in accordance with the Preferred Reporting Items for Systematic Reviews and Meta-Analyses (PRISMA) statement and the Cochrane Handbook for Systematic Reviews of Interventions. The protocol was registered in the International Prospective Register of Systematic Reviews (PROSPERO; CRD420251153720).

For reports not immediately accessible, full-text retrieval was attempted through institutional access, publisher websites, DOI/PubMed links, and library retrieval routes when available. Six reports could not be retrieved. Because eligibility could not be reliably confirmed from the available records, these reports were recorded as “not retrieved” in the PRISMA flow diagram and were not included among full-text exclusions.

### Data extraction

Two reviewers independently extracted data from included studies using a customized data extraction on a Microsoft Excel sheet. In case of disagreement, the consensus was achieved through a third reviewer.

The following data were extracted: (1) first author; (2) publication year; (3) journal; (4) study design; (5) demographic characteristics of study participants; (6) systemic therapy; (7) vaccine context (primary vaccination, booster, revaccination/catch-up, or unclear), reported pre-vaccination history for the relevant vaccine or infection, biologic or conventional disease-modifying antirheumatic drug (DMARD) exposure at vaccination, whether immunosuppressive therapy was withheld around vaccination when reported, vaccine-strain or breakthrough infection; (8) control group if available; (9) timing of post-vaccination serology or clinical follow-up; and (10) main findings.

### Quality assessment

The methodological quality of the included studies was independently assessed by two reviewers using design-specific critical appraisal tools. The randomized controlled trial was appraised using the JBI Critical Appraisal Checklist for Randomized Controlled Trials, whereas the non-randomized longitudinal studies were appraised using the JBI Critical Appraisal Checklist for Cohort Studies (Tables 1 and 2). Any disagreement was resolved through discussion with a third reviewer. The risk of bias assessment identified one randomized trial at moderate risk of bias, most cohort studies at moderate risk, and two non-randomized studies at high risk, mainly because of limited confounding control, incomplete follow-up handling, or absence of a comparator group. These judgments were considered when interpreting the certainty of the findings.

**Table 1:** Risk-of-bias assessment of the included studies using design-specific JBI critical appraisal tools: randomized controlled trial

Study	R1	R2	R3	R4	R5	R6	R7	R8	R9	R10	R11	R12	R13	Overall judgment
Heijstek et al., 2013	Y	U	Y	N	N	N	Y	Y	Y	Y	Y	Y	Y	Moderate

*R1* true randomization, *R2* allocation concealment, *R3* similarity of groups at baseline, *R4* participants blind to treatment assignment, *R5* treatment deliverers blind, *R6* outcome assessors blind, *R7* groups treated identically other than the intervention, *R8* completeness of follow-up and handling of differences, *R9* participants analyzed in the groups to which they were randomized, *R10* outcomes measured in the same way, *R11* outcomes measured reliably, *R12* appropriate statistical analysis, *R13* appropriateness of trial design and accounting for deviations. *Y* yes, *N* no, *U* unclear

**Table 2:** Risk-of-bias assessment of the included studies using design-specific JBI critical appraisal tools: non-randomized longitudinal studies (JBI cohort checklist)

Study	C1	C2	C3	C4	C5	C6	C7	C8	C9	C10	C11	Overall judgment
Bizjak et al., 2025	N	Y	Y	Y	N	Y	Y	Y	Y	N/A	Y	Moderate
Borte et al., 2009	N	Y	Y	N	N	Y	Y	Y	Y	N/A	Y	Moderate
Cakmak et al., 2023	N/A	N/A	Y	N	N	Y	Y	Y	Y	N/A	Y	High
Hamad Saied et al., 2023 (long-term immunoprotection)	Y	Y	Y	Y	N	Y	Y	Y	N	N	Y	Moderate
Hamad Saied et al., 2023 (safety follow-up study)	N/A	N/A	Y	Y	Y	Y	Y	Y	N	Y	Y	Moderate
Heijstek et al., 2007	Y	Y	Y	Y	N	Y	Y	Y	Y	N/A	Y	Moderate
Kopsidas et al., 2024	N	Y	Y	N	N	Y	Y	Y	Y	N/A	Y	Moderate
Toplak et al., 2015	N/A	N/A	Y	N	N	Y	Y	Y	Y	N/A	Y	High

*C1* similarity of groups and recruitment from the same population, *C2* exposures measured similarly, *C3* exposure measured in a valid and reliable way, *C4* confounding factors identified, *C5* strategies to deal with confounding factors stated, *C6* participants free of the outcome at study start, *C7* outcomes measured in a valid and reliable way, *C8* follow-up time reported and sufficient, *C9* follow-up complete or losses to follow-up adequately described and explored, *C10* strategies to address incomplete follow-up, *C11* appropriate statistical analysis. *N/A* not applicable when no comparison group or no attrition handling step was required. *Y* yes, *N* no, *U* unclear

In addition to the study-level judgments shown in Tables 1 and 2, we summarized the main recurring sources of bias narratively and considered them when interpreting the certainty of the evidence. Particular attention was paid to observational design, incomplete confounding control, heterogeneity of outcome definitions, incomplete follow-up, and the limited ability of small cohorts to detect rare safety events.

### Data synthesis

Because of the marked heterogeneity across studies in design, vaccine context (primary vaccination versus booster/revaccination), immunosuppressive therapy, laboratory assays, and follow-up, a quantitative meta-analysis was not considered appropriate. We therefore synthesized the data using a narrative approach, focusing on the direction and consistency of effects across studies rather than on pooled estimates.

## Results

### Study characteristics

**Fig. 1:**
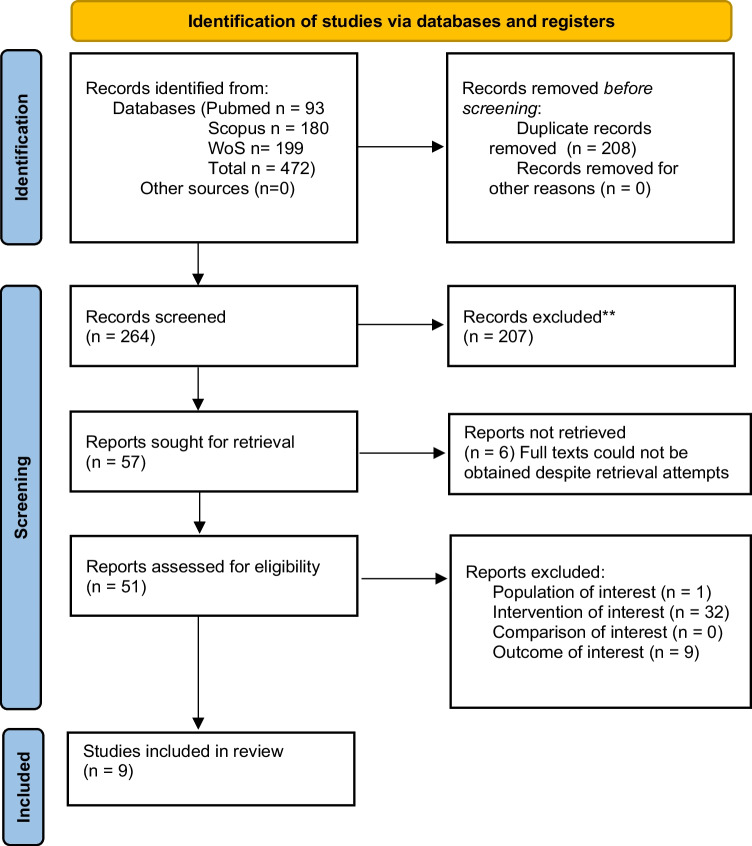
PRISMA 2020 flow diagram for new systematic reviews which included searches of databases and registers only. Source: Page MJ, et al. BMJ 2021;372:n71. https://doi.org/10.1136/bmj.n71

The electronic search identified 472 records. After duplicate removal, 264 records were screened by title and abstract. Fifty-seven reports were sought for retrieval; six full texts could not be retrieved despite retrieval attempts. Of the 51 full-text articles assessed for eligibility, 42 were excluded for the reasons shown in Fig. 1. Nine studies were included in the qualitative synthesis. The descriptive characteristics of the included studies are presented in Table 3. Five studies were retrospective, three were prospective cohort studies, and one was an open-label randomized controlled trial. Publication years ranged from 2007 to 2025. Sample size ranged from 6 to 207 patients, for a total of 743 patients with JIA. Seven studies evaluated MMR booster or revaccination, including cohorts ranging from 13 to 207 patients and one randomized trial with 63 revaccinated patients and 69 non-revaccinated JIA controls. Two small prospective cohorts assessed varicella vaccination, including 17 and 6 vaccinated children with JIA. The therapeutic background was heterogeneous and ranged from no systemic therapy to methotrexate, corticosteroids, TNF inhibitors, IL-1 and IL-6 inhibitors, abatacept, and combination regimens. Information on withholding immunosuppressive medication around vaccination was inconsistently reported: biologics were withheld before vaccination in the randomized Heijstek trial, whereas Cakmak et al. reported that biologic therapy was not interrupted.

**Table 3:** Summary of included studies by vaccine context, immunogenicity, and safety

Authors, publication year, journal	Study design	Population (M:F)	Age (years/months)	Systemic therapy	Vaccine and vaccination context	Comparison	Reviewer-extracted main outcome(s)	Secondary outcome(s)	Timepoints	Main findings in terms of primary outcome(s)	Main findings in terms of secondary outcome(s)
Bizjak et al., 2025, *Vaccine X*	Prospective	17 (M: 5; F: 12)	4.2 y (2.3–16.8)	TNFi 12, IL-6 inhibitor 4, IL-1 inhibitor 1; many combined with MTX, some tacrolimus	Varicella vaccination in VZV-naive children with JIA on selected bDMARDs; 14/17 received both first and second doses while on bDMARDs; 5 later received a third dose because of primary or secondary vaccine failure.”	Healthy controls vaccinated (*n* = 52); healthy controls after wild-type varicella (*n* = 69)	Immunogenicity of varicella vaccination in children with JIA treated with biologic therapy	Safety, tolerability, local/systemic adverse events, disease activity after immunization	1 month after each vaccine dose	Most patients (82%) developed protective antibodies after two doses, although antibody levels were lower than in healthy controls. Antibody decline over time was similar between groups	No vaccine-strain infections or serious AEs; disease activity increased in 3/17 after vaccination; 4/17 developed mild breakthrough varicella 4 months–4.5 years after vaccination
Borte et al., 2009, *Rheumatology*	Prospective	15 (sex split not reported)	6–17 y	MTX ± etanercept	Live-attenuated MMR booster	22 healthy controls	Humoral and cell-mediated immunity after MMR revaccination; effect of low-dose MTX/etanercept on vaccine efficacy	Safety: changes in disease activity and adverse events/infections	6 months before vaccine; 6 months after vaccine	MTX ± etanercept did not impair T-cell or IgG responses after MMR revaccination	No increase in disease activity or medication use within 6 months after MMR
Cakmak et al., 2023, *European Journal of Pediatrics*	Retrospective	13 JIA (M: 7; F: 6)	8–12 y	All on bDMARDs (canakinumab, tocilizumab, etanercept, adalimumab, abatacept); 6/13 also on MTX; therapy not interrupted	Live-attenuated MMR booster	None	None	Safety profile of MMR booster in children with rheumatic diseases receiving biologics	Chart review at − 6 and + 6 months; questionnaire follow-up ~ 24 months post-booster	None	Two patients had mild side effects (rash, angioedema, joint pain, fatigue). No disease exacerbation after vaccination
Hamad Saied et al., 2023, *Vaccine*	Retrospective	182 (M: 61; F: 121)	4.9 y (2.3–6.5)	No immunosuppressors 50.5%; systemic CS 3.3%; MTX 27.5%; bDMARD 6% (anti-TNF); csDMARD + bDMARD 9.3%	Live-attenuated MMR booster	None	Long-term humoral immunoprotection after MMR booster under immunomodulatory therapy	None	Antibodies measured 5 years post-booster	Five-year seroprotection in non-bDMARD vs bDMARD users: measles 86% vs 60%, mumps 94% vs 80%, rubella 83% vs 60%. Thus, 40% of bDMARD-treated children lacked protective measles and rubella antibody levels at 5 years	None
Hamad Saied et al., 2023, *Vaccine*	Retrospective	186 (M: 66; F: 120)	Age at JIA diagnosis 5.2 y (2.8–7.2)”	32% MTX monotherapy, 11% bDMARD monotherapy, 17% csDMARD + bDMARD, 37% no systemic therapy; low-dose systemic corticosteroids 1.6%	Live-attenuated MMR booster	None	None	Short- and long-term disease activity and vaccine-related adverse events after MMR booster under immunosuppressive/immunomodulatory therapies	Median visits ~ 7.3 and ~ 2.6 months pre-booster; ~ 2.7 and ~ 7.3 months post-booster	None	Mild adverse events related to the MMR booster occurred in 7% of patients. No serious adverse events were reported. Adjusted disease activity scores after MMR booster were not significantly different from pre-vaccination
Heijstek et al., 2013, *JAMA*	Prospective randomized controlled open-label trial	63 (M: 17; F: 46)	6.3 y (5.9–6.7)	MTX, NSAIDs, anti-TNF, IL-1RA, oral steroids; biologics withheld before vaccination	Live-attenuated MMR booster	JIA patients randomized to no-vaccination group (n = 69)	Immunogenicity of MMR booster in patients with JIA	Flare risk/count, safety (AEs, attenuated-virus infections), effect on disease activity	Baseline, 3 months, 12 months	At 12 months, seroprotection rates were higher in revaccinated patients than controls. Methotrexate and biologics did not affect humoral responses	Adverse events were similar between groups and unrelated to MMR. Disease activity (JADAS-27) did not differ between revaccinated patients and controls during follow-up
Heijstek et al., 2007, *Annals of the Rheumatic Diseases*	Retrospective	207 (sex split not reported)	8–10 y	NSAIDs, oral/intra-articular steroids, methotrexate, anti-TNF	Live-attenuated MMR booster	Vaccinated children aged 8–9 y (*n* = 108) vs age-matched eligible children not yet vaccinated (*n* = 86)	None	Safety of MMR vaccine: flares, medication use, disease activity, adverse events	6 months before vs 6 months after vaccination	None	No worsening of JIA disease activity after vaccination. Active joint counts, PGA, ESR, flare rate, and treatment intensity did not increase. No overt measles/mumps/rubella infections were reported
Kopsidas et al., 2024, *Rheumatology International*	Retrospective	54 (M: 4; F: 50)	2.9 y	MTX, systemic corticosteroids, intra-articular corticosteroids	Live-attenuated MMR booster	26 healthy controls	Impact of methotrexate on MMR immunogenicity	Safety after the MMR booster dose	Baseline, 12 months, 24 months	Seroprotection rates were adequate in both groups, but children with oligo-JIA on MTX appeared to have lower measles-specific IgG titers	No adverse effects, including vaccine-induced measles, during 1 month follow-up; no JIA flares for 3 months
Toplak et al., 2015, *Vaccine*	Prospective	6 (M: 2; F: 4)	4.7 ± 1.6 y	ETN/TCZ/IFX	Two-dose varicella vaccination in six stable children with JIA treated with biologic therapy; four received vaccination during biologic therapy; two received first dose three weeks before methotrexate initiation and second dose while on etanercept	None	Efficacy of varicella vaccination in children with JIA	Safety in terms of flare and adverse events	17 months	Response rate for protective antibodies after the second dose was 83%. One patient with low protective antibody levels developed mild varicella 4 months after the second dose; no serious adverse effects and disease activity remained stable; no other patients developed varicella during long-term follow-up	No serious side effects after vaccination and disease activity remained stable

*AE* adverse event, *bDMARD* biologic disease-modifying antirheumatic drug, *csDMARD* conventional synthetic disease-modifying antirheumatic drug, *JIA* juvenile idiopathic arthritis, *MMR* measles-mumps-rubella, *MTX* methotrexate, *VZV* varicella-zoster virus

Footnote(s):

Age is reported as median (IQR) unless otherwise stated. When studies reported age at JIA diagnosis rather than age at vaccination, this is specified

“Main outcome(s)” refers to the principal outcomes extracted for this systematic review; several retrospective studies did not report a formally designated primary endpoint

Booster indicates a repeat dose after prior routine vaccination. Revaccination/catch-up indicates repeat vaccination in children with absent, insufficient, or uncertain prior response/priming according to the source study

Breakthrough varicella indicates clinical varicella occurring after vaccination; this is distinct from confirmed vaccine-strain infection

### Immunogenicity of live attenuated vaccines in children with JIA

The immunogenicity evidence was dominated by MMR booster studies. In a prospective cohort, Borte et al. found that low-dose methotrexate, with or without etanercept, did not impair MMR-specific IgG or T-cell responses after revaccination [5]. Similarly, in the randomized trial by Heijstek et al., seroprotection at 12 months was higher in revaccinated children than in JIA controls who did not receive the booster, and methotrexate or biologic therapy did not materially affect the humoral response in that study [8]. Kopsidas et al. also reported adequate seroprotection after MMR booster overall, although oligoarticular JIA patients receiving methotrexate appeared to have lower measles-specific IgG titers than controls [10].

Longer-term data suggest that protection is generally maintained in many children but may be less durable during biologic exposure. Hamad Saied et al. assessed MMR-specific antibodies 5 years after booster vaccination. Seroprotection should be interpreted by vaccine component and treatment group rather than as a single overall range. Five years after booster, protective antibody levels in non-biologic disease-modifying antirheumatic drug (bDMARD) versus bDMARD users were 86% versus 60% for measles, 94% versus 80% for mumps, and 83% versus 60% for rubella. Thus, 40% of children vaccinated while receiving bDMARDs lacked protective measles and rubella antibody levels at 5 years, a finding that is not captured by an unstratified range [7].

Varicella data were more limited and should be interpreted as evidence on varicella vaccination in VZV-naive or non-immune children rather than as a booster evidence base. In the prospective case-control study by Bizjak et al., VZV-naive children with JIA receiving selected biologic DMARDs were vaccinated because they were at risk for varicella; 14 of 17 patients received both the first and second dose while on biologic therapy. Fourteen of 17 patients (82%) developed protective antibodies after the second dose, compared with 50 of 52 healthy vaccinated controls (96%). VZV-specific cellular immunity was detected in 8 of 11 patients (72%) and persisted longer than VZV-IgG, suggesting that antibody titres alone may underestimate vaccine-induced immunity. Five patients later received a third dose because of primary or secondary vaccine failure [4]. Toplak et al. assessed a two-dose varicella vaccination schedule in six stable children with JIA treated with biologic therapy; five of six developed protective antibodies after the second dose, whereas one child with low protective antibody levels later developed mild varicella 4 months after the second dose [12].

Overall, the included studies support satisfactory short-term immunogenicity of live attenuated vaccines in clinically stable children with JIA, especially in the booster setting. At the same time, they suggest that methotrexate and biologic therapy may influence the magnitude or persistence of protection in selected patients, particularly for measles and varicella [4, 7, 10, 12].

### Safety of live attenuated vaccines in children with JIA

Safety outcomes were interpreted across three domains: disease activity or flare, local/systemic adverse events, and attenuated-virus or breakthrough infection. Across MMR studies, no serious vaccine-related adverse events and no vaccine-induced measles, mumps, or rubella infections were reported where these outcomes were assessed. Mild adverse events were reported in a minority of patients, including 7% in the Hamad Saied safety follow-up study. Disease activity measures, including JADAS/cJADAS or individual components such as active joint count, physician global assessment, ESR, and treatment intensity, did not show a consistent post-vaccination worsening [5, 6, 8–11].

For varicella, Bizjak et al. reported no vaccine-strain infections or serious adverse events, but disease activity increased in 3 of 17 patients after vaccination and 4 of 17 developed mild breakthrough varicella 4 months to 4.5 years after vaccination [4]. Toplak et al. reported no serious adverse effects and stable disease activity, although one child with low antibody levels developed mild varicella 4 months after the second dose [12]. These findings support a reassuring safety profile but do not exclude rare adverse events because varicella cohorts were small.

## Discussion

The available evidence on live viral vaccination in juvenile idiopathic arthritis is clinically reassuring but remains limited and heterogeneous. Across the included studies, MMR booster/revaccination and varicella vaccination were generally associated with acceptable immunogenicity and a low frequency of mostly mild adverse events, without a consistent signal of sustained flare induction or clinically relevant worsening of disease activity. However, the strength of this conclusion differs substantially between vaccines and outcomes.

The evidence is strongest for MMR booster vaccination. One randomized controlled trial and several cohort studies support the short-term safety and immunogenicity of MMR booster/revaccination in clinically stable children with JIA, including many receiving methotrexate and selected biologic therapies. This broadly supports the EULAR/PRES recommendation that MMR booster vaccination may be administered to patients receiving methotrexate and may be considered in selected patients receiving low-dose glucocorticoids, TNF inhibitors, IL-1 inhibitors, or IL-6 inhibitors. Nevertheless, our review also shows why this recommendation should remain individualized rather than automatic: the available studies are not powered to detect rare safety outcomes, treatment interruption was inconsistently reported, and long-term humoral protection may be lower in children vaccinated while receiving biologic therapy.

The clearest example is the 5-year MMR seroprotection study by Hamad Saied et al. When reported by vaccine component and treatment group, seroprotection among non-bDMARD versus bDMARD users was 86% versus 60% for measles, 94% versus 80% for mumps, and 83% versus 60% for rubella. Measles deserves particular emphasis because of its transmissibility and epidemiological relevance and because the between-group difference reached statistical significance. However, the rubella finding is also clinically relevant, since 40% of bDMARD-treated children lacked protective rubella antibody levels at 5 years. These data suggest that the key residual issue after MMR booster is not short-term safety but durability of protection in selected biologic-treated children. In practice, this supports considering vaccination before escalation to biologic therapy when feasible, and post-vaccination serology in selected higher-risk patients, particularly when vaccination occurred during biologic treatment or when exposure risk is high.

Available data are insufficient to draw robust drug-class-specific conclusions. Most biologic-exposed MMR data are driven by TNF inhibitors, while smaller numbers of patients received IL-1 inhibitors, IL-6 inhibitors, abatacept, or combination regimens. Therefore, the present review should not be interpreted as proving equivalent vaccine response across biologic classes. Rather, it suggests that MMR booster/revaccination can be considered in selected stable patients under specialist supervision, while acknowledging that the magnitude and persistence of humoral protection may vary and require individualized follow-up.

The varicella evidence base is smaller and should be interpreted more cautiously. The two prospective cohorts mainly inform varicella vaccination in VZV-naive or non-immune children with stable JIA, including biologic-treated patients, rather than routine booster vaccination. Bizjak et al. showed that most patients developed protective antibodies after two doses, but antibody levels were lower than in healthy controls and mild breakthrough varicella occurred in some patients during follow-up. Importantly, VZV-specific cellular immunity was detectable in most tested patients and persisted longer than VZV-IgG, suggesting that antibody titers alone may not fully capture vaccine-induced protection. This finding complicates post-vaccination monitoring: serology is clinically practical, but absence or decline of detectable antibodies may not necessarily mean absence of immune protection.

Safety findings were reassuring but should be interpreted in relation to sample size and outcome ascertainment. Across MMR studies, serious vaccine-related adverse events and vaccine-induced infections were not reported where assessed, and disease activity generally remained stable. For varicella, no vaccine-strain infection was reported, but breakthrough varicella occurred in a minority of vaccinated patients and was mild. These data support specialist-guided vaccination in stable patients, but they do not eliminate the possibility of rare events, particularly because varicella cohorts were small.

The risk of bias assessment should temper the interpretation of the findings. Apart from one open-label randomized trial, most studies were observational, several had limited or incomplete control for confounding, and outcome definitions, laboratory assays, treatment exposures, and follow-up intervals varied substantially. In Tables 1 and 2, most studies were judged at moderate risk of bias and two non-randomized studies at high risk of bias. These limitations reduce certainty and support cautious language in the conclusions.

Despite these limitations, the clinical relevance of the findings is high. Children with JIA receiving immunosuppressive therapy remain vulnerable to vaccine-preventable infections, and reliance on herd immunity may become less reliable when community vaccination coverage declines or outbreaks occur. The present findings therefore support a pragmatic approach: assess live viral vaccination before treatment escalation whenever possible, consider vaccination in clinically stable patients under pediatric rheumatology supervision, document treatment exposure and vaccine history carefully, and use post-vaccination serology selectively when the risk of waning or incomplete protection is clinically meaningful.

Future studies should prioritize standardized prospective data collection, but large long-term observational registries may be more feasible than dedicated trials for rare safety outcomes. Such registries should capture vaccine type and dose, primary versus booster/revaccination context, timing relative to immunosuppressive therapy, medication withholding, disease activity, vaccine-strain and breakthrough infections, antibody and cellular immunity where possible, and long-term clinical outcomes.

## Conclusions

In children and adolescents with juvenile idiopathic arthritis, the available evidence on live viral vaccination mainly concerns MMR booster/revaccination and, to a lesser extent, varicella vaccination in VZV-naive or non-immune children. In clinically stable patients assessed by pediatric rheumatology specialists, these vaccines appear generally immunogenic and clinically safe, including in many children receiving methotrexate or selected biologic agents. However, the evidence remains limited by small sample sizes, predominantly observational designs, heterogeneous treatment exposures, and incomplete confounding control. The main residual concern is incomplete or less durable protection in selected biologic-treated patients, particularly for measles and rubella 5 years after MMR booster and for varicella in low responders. These findings support individualized vaccination decisions, preference for vaccination before biologic escalation when feasible, and consideration of post-vaccination serology in selected higher-risk children.

## Supplementary Information

Below is the link to the electronic supplementary material.

ESM 1 (DOCX 2.17 MB)

## Data Availability

All data analyzed in this systematic review are included in this published article and its referenced sources.

## Abbreviations

AE: Adverse event
bDMARD: Biologic disease-modifying antirheumatic drug
cJADAS: Clinical Juvenile Arthritis Disease Activity Score
csDMARD: Conventional synthetic disease-modifying antirheumatic drug
ESR: Erythrocyte sedimentation rate
IgG: Immunoglobulin G
IL-1: Interleukin-1
IL-6: Interleukin-6
JADAS: Juvenile Arthritis Disease Activity Score
JIA: Juvenile idiopathic arthritis
MMR: Measles-mumps-rubella vaccine
MTX: Methotrexate
PRISMA: Preferred Reporting Items for Systematic Reviews and Meta-Analyses
PROSPERO: International Prospective Register of Systematic Reviews
RCT: Randomized controlled trial
TNF/TNFi: Tumor necrosis factor/tumor necrosis factor inhibitor
VZV: Varicella-zoster virus
