# Supplementary material for: Live viral measles-mumps-rubella revaccination and varicella vaccination in children and adolescents with juvenile idiopathic arthritis: a systematic review
Source: Eur J Pediatr. 2026 Jun 16;185(7):505. doi: 10.1007/s00431-026-07160-6 (PMC13269145; doi:10.1007/s00431-026-07160-6)
Supplement: Supplementary file 1 — (DOCX 2.17 MB) [file 431_2026_7160_MOESM1_ESM.docx]

Supplementary Table S1. Search strategy

| -Pubmed: (juvenile idiopathic arthritis[MeSH] OR juvenile idiopathic arthritis[tiab] OR JIA[tiab])AND (vaccin*[tiab] OR immunization[tiab] OR immunisation[tiab] OR influenza vaccines[MeSH] OR live attenuated vaccines[MeSH] OR measles-mumps-rubella vaccine[MeSH] OR varicella vaccine[MeSH]) AND (immunogenic*[tiab] OR seroprotection[tiab] OR seroconversion[tiab] OR antibody*[tiab] OR neutralization[tiab] OR avidity[tiab] OR cellular immunity[tiab] OR T-cell[tiab] OR safety[tiab] OR adverse[tiab] OR flare*[tiab] OR disease activity[tiab]) AND (child[MeSH] OR adolescent[MeSH] OR pediatr*[tiab] OR paediatr*[tiab]) NOT (letter[pt] OR editorial[pt] OR "conference abstract"[Publication Type]) |
| --- |
| -Scopus: TITLE-ABS-KEY(("juvenile idiopathic arthritis" OR JIA) AND (vaccin* OR immunization OR immunisation OR "measles-mumps-rubella vaccin*" OR "measles mumps rubella vaccin*" OR "varicella vaccin*") AND (immunogenic* OR seroprotection OR seroconversion OR antibody* OR neutralization OR neutralisation OR avidity OR "cellular immunity" OR "T-cell*" OR "T cell*" OR safety OR adverse OR flare* OR "disease activity") AND (child* OR adolescent* OR pediatr* OR paediatr*)) AND (DOCTYPE(ar) OR DOCTYPE(re)) |
| -Web of science: ("juvenile idiopathic arthritis" OR JIA) AND (vaccin* OR immunization OR immunisation OR "measles-mumps-rubella vaccin*" OR "measles mumps rubella vaccin*" OR "varicella vaccin*") AND (immunogenic* OR seroprotection OR seroconversion OR antibody* OR neutralization OR neutralisation OR avidity OR "cellular immunity" OR "T-cell*" OR "T cell*" OR safety OR adverse OR flare* OR "disease activity") AND (child* OR adolescent* OR pediatr* OR paediatr*) |
